# Lugol’s solution and other iodide preparations: perspectives and research directions in Graves’ disease

**DOI:** 10.1007/s12020-017-1461-8

**Published:** 2017-10-26

**Authors:** Jan Calissendorff, Henrik Falhammar

**Affiliations:** 1 Department of Endocrinology, Metabolism and Diabetes, Karolinska University Hospital, Stockholm, Sweden; 2 Department of Molecular Medicine and Surgery, Karolinska Institutet, Stockholm, Sweden

**Keywords:** Hyperthyroidism, Thyroidectomy, Iodide, Adjuvant treatment, Escape

## Abstract

Lugol’s solution and other preparations containing iodide have for almost a century been used as an adjuvant treatment in patients with Graves’ disease planned for thyroidectomy. Iodide has been shown to decrease thyroid hormone levels and reduce blood flow within the thyroid gland. An escape phenomenon has been feared as the iodide effect has been claimed to only be temporary. Lugol’s solution has many additional effects and is used in other settings beside the thyroid. Still, there are questions of its mode of action, which doses should be deployed, if it should be used preoperative in all thyroidectomies or only in a few selected ones if at all, what is its use in other forms of thyrotoxicosis besides Graves’ disease, and what is the mechanism acting on the vasculature and if these effects are confined only to arterial vessels supporting the thyroid or not. This review aims to collate current available data about Lugol’s solution and other iodide preparations in the management of Graves’ disease and give some suggestions where more research is needed.

## Introduction

Lugol’s solution (LS) was developed 1829 by the French physician Jean Guillaume August Lugol, initially as a cure for tuberculosis. It is a solution of elemental iodine (5%) and potassium iodide (KI, 10%) together with distilled water. It has been used as a disinfectant, a reagent for starch detection in organic compounds, in histologic preparations, in dental procedures and in diagnosis of cervical cell alterations, the Schiller´s test (Table 1). Already in the 1920s LS was given as a pre-treatment to thyroid surgery [1]. By that time LS became the standard pre-operative treatment in patients with Graves’ disease (GD). Iodide treatment could also be given as a saturated solution of potassium iodide (SSKI) or tablets. Radiopaque cholecystographic agents such as iopanic acid containing iodide has also been used previously, although nowadays their use is restricted [2]. These agents are also potent inhibitors of type 1 and type 2 deiodinases, blocking the conversion of T4 to T3 and rT3 to T2 [3].

**Table 1:** Examples of usage of Lugol’s solution and other iodine preparations

Graves’ disease
Disinfectant
Sterilisation
Foot ulcers
Dental procedures
Histopathology
Schiller´s test
Water purification
Radiation emergency
Iodine deficiency/ cretinism
Protection from goiter

Iodide is the ion state of iodine which is the result when elemental iodine (which is corrosive) binds to e.g. potassium and in this state can be more easily consumed or applied topically. However, often the terms iodide and iodine are used interchangeably. With the development of pharmacologic agents such as antithyroid drugs and with radioiodine therapy, LS is not routine anymore in many countries [4–6], although it is still advocated in current American Thyroid Association guidelines [7], although this has recently been challenged [8].

GD is a common autoimmune disease, with typical symptoms as weight loss, heat intolerance, tachycardia and mental disturbances as tiredness and restlessness. There is a female preponderance (4:1 in a first episode of hyperthyroidism) with a peak incidence at 40–69 years [9]. Antithyroid drugs are often chosen since these are mostly well-tolerated, and can induce cure in around 50% after 12–18 months of treatment [10]. These pharmacologic compounds, propylthiouracil, methimazole or carbimazole, block the thyroid hormone synthesis by inhibiting thyroid peroxidase. In cases of large goiters, intolerance to medication or recurrent disease, surgery or radioiodine treatment could be alternative treatment. Radioiodine therapy, inducing life-long hypothyroidism, has been the preferred first option in most US centers [11], while in European centers antithyroid drugs are preferred [12]. Surgery is often a secondary option performed with subtotal (<2 g), near total or total thyroidectomy to minimize the risk of recurrence. To diminish the risk of vascular complications hyperthyroidism should be pre-treated before operation [7]. This could be by beta-blockers alone, or together with antithyroid drugs, with or without adding LS. Moreover, LS could also be used when there is a need to control GD promptly to avoid serious consequences [4, 13].

The aims of this review are to provide a summary of currently available knowledge of LS including other iodide preparations in the treatment of GD and give some suggestions in areas where more research is needed.

## Iodide effects in healthy man and in rats

Iodide (I^−^) is essential for the synthesis of thyroid hormones, T4 and T3. The primary source is food, often salt fortified with iodine. The recommended daily intake is 150 μg in adults, with a preferred increase in pregnant and lactating females [14]. Iodide is transported across the basement membrane by the Na^+^/I transporter where it is rapidly oxidized by H_2_O_2_ to iodine, catalyzed by thyroid peroxidase and is then incorporated into thyroglobulin. In healthy humans who are not predisposed to thyrotoxicosis, increased thyroid hormone synthesis can be induced by high doses of iodine such as iodine containing contrast agents, and after a long-time use of antiseptics such as povidone–iodine swabs [15], in dietary supplements as kelp, and secondary to iodine containing medications such as amiodarone. Radiographic contrast media and amiodarone can contain much more iodine than daily requirements, 75 mg and 320–370 mg, respectively. The thyroid had been estimated to tolerate up to 1100 μg/iodine/day [14]. By an iodide overload hyperthyroidism could ensue as a consequence [16, 17], the Jod-Basedow effect, which is more frequent in iodine deficient areas, and in patients with multinodular goiter. Iodine excess can also lead to other untoward thyroid complications, such as iodide goiter, iodide-induced hypothyroidism, and iodide-induced subacute thyroiditis [16, 18, 19]. These pathologic consequences will disappear when iodine is withdrawn [20]. Symptom relief from iodine induced thyrotoxicosis can be achieved with beta-blockers and in severe cases with high doses of antithyroid drugs [7, 21].

## Mechanisms of iodide on the thyroid gland and the escape phenomenon

In the short term LS reduces the thyroid hormones, T4 and T3 by increasing iodine uptake and inhibiting the enzyme thyroid peroxidase [22], thus attenuating oxidation and organification of thyroid hormones [23]. Moreover, the release of thyroid hormones is also blocked [24, 25]. The mechanism could partly be explained by activation of substances as iodolactones and iodoaldehydes as these have been shown to inhibit nicotinamide adenine, dinucleotide phosphate oxidase, thyroid peroxidase, and TSH-induced cyclic adenosine monophosphate (cAMP) formation in the thyroid [26].

Wolff-Chaikoff is the effect of iodide in normal mice which lead to an increase of intrathyroidal iodine concentration within 24–48 h and a subsequent decrease of thyroid hormone synthesis [27]. In healthy subjects there is an adaption to iodine excess by an autoregulatory mechanism within the thyroid, which serves as a defense against fluctuations in the supply of iodine and permits escape from the paradoxical inhibition of hormone synthesis that a very large quantity of iodine induces. Defective or absent autoregulation can occur in predisposed patients, as in those with euthyroid Hashimoto’s thyroiditis and in GD-patients treated with radioiodine or subtotal thyroidectomy [28]. Thus, these are more prone to develop hypothyroidism secondary to an iodine overload. Hyperthyroidism more commonly occurrs in iodine deficent subjects, and in patients with multinodular goiter.

The escape from acute Wolff-Chaikoff effect is associated with a decrease in thyroid sodium/iodide symporter causing a reduction in intrathyroidal iodide concentration [29]. There is also a form of escape following iodide therapy in GD which has been described as common [30, 31]. Thus, in treating patients with hyperthyroidism with LS an exacerbation of thyroid hormone levels could be a consequence after a period of blocking the thyroid, as the gland has become loaded of iodine substrate for hormone synthesis [32].

However, in the investigation by Takata et al. a combination of iodide solution was used together with methimazole for up to 8 weeks [33]. Iodide was discontinued when patients showed normal free T4. Eleven patients (25%) escaped from the Wolff-Chaikoff effect, and 3 derived no benefit at all. Moreover, in another study including patients with mild GD who received primary treatment with LS (50–100 mg daily), control of hyperthyroidism after 12 months was comparable with that seen in patients receiving low-dose methimazole treatment [34]. How often and how early escape occurs is not clear, but in an observational study from Japan long-term treatment with LS alone or in combination with antithyroid drugs has been used, with 29/44 (66%) being well-controlled on 100 mg LS daily alone for 7 years [35]. In another study of 21 patients with hyperthyroidism given iodide daily, hormone levels started to increase again after 3 weeks in some, but others remained euthyroid even after 6 weeks [36]. Reactivation of thyrotoxicosis could to some extent be explained by a stimulation of the immune system as elevation of TSH receptor antibodies has been noted in euthyroid patients preoperatively with 60 mg iodide twice daily for 10 days [37]. However, in long-term treatment with iodide these antibodies has been reported to decline [35].

## Vascular effects

Plummer observed a 75% decrease in mortality associated with thyroidectomy when LS was introduced [1]. At that time metabolic rate decreased as well as symptoms. As a complement to effect on T4 and T3 reductions vascular effects has been of interest and already in 1925 intrathyroid blood vessel compression was described after LS therapy [38]. Reduced blood flow has since then been described as an effect of LS in GD patients with different methods. In 9 subjects with GD uptake of thallium was decreased with a third after 10 days of LS (0.5 ml tds) [39], and the authors speculated that this could be a result of decreased perfusion, as the amount of colloid had increased. A reduction in vascularity measured with ^99m^TC-pertechnetate after LS has also been shown [40]. In a number of investigations of euthyroid individuals with GD LS has reported to decrease the rate of blood flow, thyroid vascularity, and intraoperative blood loss during thyroidectomy [39, 41–44].

In a recent randomized control trial in patients receiving LS median blood losses (50 vs. 140 mL), and operative times (138 vs. 150 min), were also significantly less compared to controls [45]. The reduced blood loss is associated with both a 60% reduction in systemic angiogenic factor (VEGF) and with 50% of interleukin-16 [46]. If other angiogenic mediators also are involved is unknown. Furthermore, microvessel density, calculated with ultrasound, displayed decreased blood flow after 10 days of 10 drops iodide (74.7 vs. 54.4, mL/min), decreased blood loss (128.6 vs. 108.7 mL) and less expression of CD34 measured with immunohistochemistry [42]. On the other hand, another study demonstrated no difference in blood loss or time of surgical procedure comparing 13 patients on iodide vs. 24 on antithyroid drugs [47].

## Treatment with LS

LS tastes bitter and is also corrosive, and this should be disguised by taking it with a sweet drink such as apple juice. The applied doses come from experience rather than by prospective randomized controlled trials (Table 2). Historically Plummer used 80–320 mg iodide daily and this was established as a pre-treatment before thyroidectomy in GD [1]. However, the efficacy of much smaller doses has also been investigated in the late 1920s to 1960, also in long-term treatment. Thomson et al. found that 6 mg daily iodine induced euthyroidism in most patients [48], and as low doses as 1 mg has also been effective [49]. Reports on escape and the development of antithyroid drugs and radioactive therapy then discouraged physicians from this route of treatment.

**Table 2:** Examples of different applied doses of iodide and preparations in treatment of Graves’ disease in different publications

Publication	Lugol’s solution 5–8 mg/drop	SSKI 50 mg/drop	Tablet KI	Iodide mg/day	Treatment duration	Number of patients with iodide
Plummer [1]	10 drops od to qid			80–320	10 days	600
Feek 1980 [54]			60 mg tds	?	10 days	10
Roti 1988 [57]		6 drops bd		456	10 days	8
Kaur 1988 [6]	0.4 ml tds			15	10 days	24
Tan 1989 [51]	0.5 ml daily			50	10 days	10
Philippou 1992 [36]	10 drops tds			114	3–7 weeks	21
Erbil 2007 [42]	10 drops tds			114	10 days	17
Takata 2010 [33]			50 mg daily	38.2	4.9 ± 3.8 weeks	32
					6.2 ± 3.1 weeks^a^	37
Uchida 2014 [34]			50–100 mg daily	35–75	1 year	30
Okamura 2014 [35]			50 mg daily	10–800	Years	44
Sato 2015 [50]			50 mg daily	38.2	up to 90 days	161
Yilmaz 2016 [43]	0.8 mg/kg			56^b^	10 days	20
Fischli 2016 [52]	13 drops tds			243.75	10–14 days	10
Calissendorff 2017 [4]	5 drops tds			100.5	7–10 days	27
Ross 2016 [7]	5–7 drops tds			40–56	10 days	
(ATA Guidelines)		1–2 drops tds		50–100		

*SSKI* saturated solution of potassium iodide

^a^ With addition of 30 or 15 mg methimazole daily, longer iodide treatment in the group receiving low dose methimazole

^b^ Dose in a 70 kg man

Takata et al. demonstrated that normalisation of free T4 was more rapid with a combination of LS and antithyroid drugs, than methimazole alone, however, remission did not differ during a 4–5 year follow-up [33]. In another Japanese study the combination also resulted in a higher propotion of normalized thyroid hormones at 30 and 60 days compared to antithyroid drugs alone [50]. In both these investigations iodide was stopped when free T4 had normalized. In the ATA guidelines 5–7 drops thrice daily of LS or 1–2 drops of SSKI (50–100 mg iodide) 10 days before thyroidectomy is recommended. SSKI contain 1 g of potassium iodide per ml. An iodide dose of 5 drops LS thrice daily is equivalent to 100.5 mg iodide daily [4]. Yilmaz et al. used twice as high dose in their study [43]. Other doses of iodide have also been applied such as 150 or 375 mg daily [36, 51], all with good effect in reducing free T4 and free T3. Lower doses as 50 mg KI have also been applied (equivalent to 38.2 mg of iodide) [50]. In long-term treatment 10–400 mg iodide has been used, with 40% experienced remission on iodide alone during a follow-up of 17.6 years (range 8.6–28.4) [35]. Thus, the doses used vary considerably and which dose has the most favourable vascular effects are unknown.

In uncontrolled GD, or if adverse effects of antithyroid drugs develops such as agranulocytosis or liver failure, rescue treatment with LS is one option. Radioactive iodine could also be deployed but the fear of thyroid storm if thyroid function exacerbates by this therapy could make LS followed by thyroidectomy a reasonable choice. We recently demonstrated that LS in this setting was effective and decreased both thyroid hormone levels and heart rate with few side effects [4]. In a Swiss investigation, patients with high free T4 and free T3 were pretreated before surgery with betablockers, 2 mg dexamethasone (to inhibit peripheral conversion to T3) and 13 drops LS thrice daily (243.75 mg of iodide daily) for 10–14 days, and acquired almost normalization of thyroid hormones [52].

## Iodide compounds and iodine content

There are several iodide compounds available. Classically LS is prescribed in a dose of 5–10 drops thrice daily with each drop containing 5–8 mg/drop [7, 42], or 1–2 drops SSKI, 50 mg/drop thrice daily. Higher doses of SSKI has also been used [53]. Iodide tablets, if available, containing 60 mg has been given as three times daily [54]. In Japan, tablets with 50 mg potassium iodide (KI) have been used [34, 35, 50]. When examining the iodide content in 5% dental solution (iodine and iodide potassium) the iodide content was 6.7 mg/drop equivalent to LS [4]. Lower doses have been subscribed when long-term treatment is planned, rather than as a pre-operative adjunct [33, 48–50].

## Adverse effects

LS and other iodide preparations seem to have low frequency of adverse effects. In doses of 1000 times the normal nutritional need, side effects may include: acne, loss of appetite, or upset stomach. More severe side effects are fever, weakness, unusual tiredness, swelling in the neck or throat, mouth sores, skin rash, nausea, vomiting, stomach pains, irregular heartbeat, numbness or tingling of the hands or feet, or a metallic taste in the mouth. (www.medicinenet.com/potassium_iodide-oral/article.htm Accessed 10 August 2017). In the study by Sato et al. adverse effects with a combination of methimazole 15 mg and inorganic iodine (KI tablets) at dose of 38.2 mg per day were less common than in a group of patients treated with methimazole alone with 30 mg daily [50]. We found that 15% of our patients treated with LS experienced mild adverse effects, mostly in form of rash [4]. Okamura et al. studied patients with GD with adverse effects to antithyroid drugs who were switched to KI tablets instead [35]. The only adverse effects found were a transient increase in TSH receptor antibodies and hypothyroidism in a few patients. However, an anaphylactic-like reaction has been reported when using LS in diagnosis of cervical cell alterations (Schiller’s test) [55]. Other adverse effects such as iodide-associated sialadenitis (iodide mumps) after iv iodide contrast [56], and esophageal ulcers have also been described. Allergic reactions to iodine compounds are possible, and LS treatment in patients with dermatitits herpetiformis should be performed with caution as this is linked to iodine sensitivity (www.fda.gov/downloads/Drugs/GuidanceComplianceRegulatoryInformation/Guidances/UCM080542.pdf Accessed 10 August 2017).

## Future research directions

Most studies on LS therapy have been retrospective and often with few participants. Recently the issues on blood loss and vasculature effects were questioned due to the lack of proper randomized trials [8]. However, we have here made a comprehensive review of the literature on iodide therapy, doses to be applied, on effects on the vasculature, and on short and long-term treatment, also discussing the escape phenomenon, reviewing limitations and current insights.

There is a need for future prospective investigations with a sufficient number of participants but also randomized control trials as well as in patients with mild vs. severe GD. This would enable us to draw firm conclusions if LS, alone or in combination with antithyroid drugs, do affect operative time, blood loss, and surgical complications such as hypocalcemia and damage of the recurrent laryngeal nerve. The efficacy of long-term treatment with LS needs also to be further explored. It is not proven if the iodine background, ethnicity or other factors make the relatively low number of patients escaping from iodide blocking in Japan a phenomenon which should not be feared to the same extent as it has in the past. To explore this investigations in areas with different iodine backgrounds have to be performed with focus on control of GD, degree of escape from therapy and adverse effects. Adverse events need to be better recorded, both in the short and long-term use of LS therapy. The optimal dose of LS also needs to be clarified. Moreover, the mechanisms of LS, especially on the vasculature are intriguing and merit further research. It is fascinating that almost 200 years since this compound was invented, and 100 years since it became a treatment in GD, we still do not fully understand how the effects on the vasculature are mediated. We also do not know if the vasculature effects in GD are present in other thyroidal diseases such as goiter or multinodular diseases, or in other vascular beds in the body.

## Conclusion

LS has been advocated for almost 100 years in the treatment of GD. It has effects in decreasing thyroid hormones, and possibly in reducing blood flow during thyroidectomy. LS is used both in combination with antithyroid drugs preoperatively in planned thyroidectomies in certain centers routinely, and alone as a rescue therapy if severe side effects to antithyroid drugs have occurred. These effects, especially on the vasculature have to explored further, together with investigations on how common there is an escape from LS-blocking, the mechanisms of action, which doses that should be applied, and registration of side effects.
